# Supplementary material for: The probability of Plasmodium vivax acute illness following primary infection and relapse in Papua New Guinea
Source: PLoS Negl Trop Dis. 2025 Oct 3;19(10):e0013567. doi: 10.1371/journal.pntd.0013567 (PMC12510656; doi:10.1371/journal.pntd.0013567)
Supplement: S1 Appendix — (DOCX) [file pntd.0013567.s001.docx]

**S1 Appendix: Simulation model**

We require the expected numbers of primary infections and relapses in the cohort. The simulation model is required in order to take previous estimates of the forces of infection from primary infection and relapse in the cohort and adjust them for treatment and for the different relapse definitions. (The previous estimates of the relapse rates would be equivalent to relapse definition A with no masking by treatment). This simulation includes components for inoculation, primary infection, the number and timing of relapses, blood-stage clearance, clinical illness and treatment. Each component is parameterized using statistical analyses of the cohort in Papua New Guinea or other data sources from the same geographical area. The simulation model uses five-day time-steps, $s$, which can be aggregated to match the two-month intervals, $t$, of the cohort study.

We include two sets of input parameter values for the simulation model, arising from uncertainty in the duration of blood-stage infections. Ensemble modelling has previously been used to reflect uncertainty in climate modelling and models of *P. falciparum* malaria [1-3].

This model is solely focused on *P. vivax* dynamics; *P. falciparum* infections are not included.

*(i) Primary infection and the duration of blood-stage infection*

The incidence and seasonal pattern of primary infection were estimated using genotyping data from the Ilaita cohort in our earlier work [4]. We use our previous assumptions to build the present model component for primary infection.

Briefly, we assumed that the seasonality of *P vivax* primary infection is similar to that of *P falciparum* since they are transmitted by the same vectors and, because *P falciparum* does not have the ability to relapse, the seasonality could be estimated. We assumed a repeating seasonal pattern for primary infections. The previous estimates also accounted for both imperfect detection and treatment.

In our previous work, we used fixed values for the mean duration of blood-stage infection (in the absence of treatment) in order for the estimates of the force of primary infection to be identifiable. These came from two sources, and we included both to reflect the range of uncertainty. Therefore, we form two sets of parameter values to input into the current model. The shorter estimate of 27 days was informed by a study estimating the mean durations of blood-stage infection in a cohort of children in endemic areas in Albinama, Papua New Guinea and in Thailand [5] (input parameter set 1). The longer duration comes from neurosyphilis patients deliberately infected as malaria therapy in the 1940s and 1950s. Among those infected for the first time with *P. vivax* via blood, a mean of 76 days was used (input parameter set 2). We did not include an effect of acquired immunity on the duration of blood-stage infection. Little is known for *P. vivax*. For *P. falciparum*, acquired immunity has been found not to shorten the duration of blood-stage infection [6, 7]. Although one explanation to reconcile the two different estimates of blood-stage infection duration would be that the different biology of *P. falciparum* and *P. vivax* could lead to different effects and the duration decreases with exposure, another may be that the *P. vivax* malaria therapy strains are not be representative [8].

The force of primary infection was estimated to be 13.3 (95% CI 11.9, 14.2) per year for parameter set 1 for a three year old child in Ilaita village who did not use insecticide-treated nets (ITNs), and 11.5 (10.5, 12.3) for parameter set 2. The associations with village and ITN use were previously estimated. For participants using ITNs for 50% or more of nights compared to less than 50%, a rate ratio of 0.70 (0.63, 0.77) was estimated. For Sunita village compared to Ilaita village, the rate ratio was estimated to be 1.16 (1.04, 1.28).

In both our earlier work and the current simulation, we assume that the force of primary infection varies by age proportional to body surface area. We obtained the median height and weight for the mid-point of each one-month age-group from the WHO growth standards [9] and calculated body surface area using the formula of Mosteller [10] (Fig). We do not take sex into account in the simulation and use the median measurements for girls for both sexes. Within each month of age, the body surface area was assumed to be constant.

| Fig. Ratio of body surface area by age to that of a three year old girl using WHO growth standards [9] |
| --- |
| 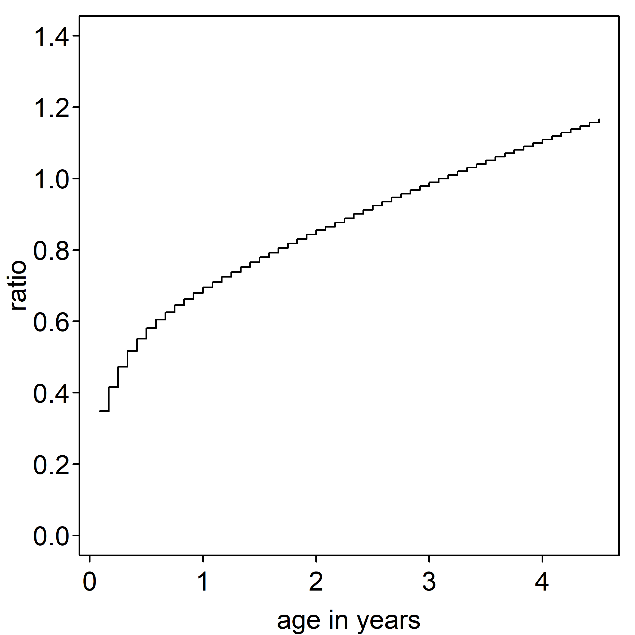 |
|  |

In the current model, the number of primary infections $m(a,s)$ for an individual in covariate (age-group, village and ITN-use) category $a$ introduced in five-day time-step $s$ follows a Poisson distribution around the incidence rate $\lambda\left( a,s \right)$ so that $m(a,s) \sim Poisson(\lambda(a,s))$. The incidence of primary infection was estimated in two-month intervals in the cohort study. All five-day time-steps, $s,$ within the same two-month interval $t$, are assumed to stem from the same value of $\lambda\left( a,s \right)$.

*(ii) Treatment*

The simulated effect of treatment was to clear blood-stage infections arising from both relapses and primary infection. The blood-stage infections were truncated at the time of administration, and new blood-stage infections were prevented from establishing for a fixed prophylactic period of 14 days. Treatment was assumed to be 100% efficacious based on estimates of early treatment efficacy [11].

The treatment rates used in the simulation model were estimated from the cohort data. Blood-stage antimalarial treatments were given following *P. vivax* and *P. falciparum* illness by the study team, and additionally outside the study. The treatment rates specific to each two-month time interval, age-group, village and ITN use combination were estimated from the observed number of treatments and the number of children.

Although the dates when the treatments were given are recorded, the timing of the individual primary infections and relapses is not known since these are estimated for the aggregated covariate category. Therefore the timing of treatment in relation to the blood-stage infection is unknown. In the simulation, treatment timing was assigned to individual children stochastically. We recognize that our assumption of a constant rate independent of infection dynamics is a simplification since a proportion of the treatments will be in response to illness from *P. vivax,* as well as *P. falciparum* and non-malarial fevers.

The rate of treatment for covariate category $a$ and time-step $s$ is represented by $q(a,s)$. We assume that the treatment rates before the study period are the same as those during follow-up. Sensitivity analyses indicated that this assumption did not greatly affect the estimates.

*(iii) Number of viable hypnozoites per brood*

Each infection $i$ is assigned a number of viable hypnozoites or relapsing non-circulating merozoites, $h_{i}$. We define the distribution for $h_{i}$using a rule found to hold for several strains [12]. The rule states that given a proportion $d$ of primary infections with at least one relapse, then the proportion of primary infections with up to $h_{i}$ relapses is $d^{h_{i}}$. Since we cap the number of allowed relapses at a maximum of 15 rather than infinite, the resulting distribution is rescaled so that the probabilities sum to one. Heterogeneity in relapse rates between individuals has also been observed elsewhere [13].

The previous analysis of the Ilaita cohort estimated the mean number of relapses per primary infection to be 5.9 (5.6, 6.2) for parameter set 1 and 4.3 (4.0, 4.6) for parameter set 2 [4]. These correspond to values of $d$ of 0.926 (0.911, 0.939) and 0.846 (0.828, 0.861), respectively. These estimates are similar to those found in another modelling study using different methods (4.7) [14], estimates from a cohort of primaquine-treated children and controls [18] and findings from a study in US volunteers [12, 15].

In the simulation, the number of relapses is determined when the primary infection is assigned. A child can have several broods in their liver at the same time, with each acting independently.

*(iv) The distribution of the time to relapse*

Each viable hypnozoite is randomly assigned a time of relapse from a distribution, $f_{h}(x)$. We assume that the timing of relapses is independent from other broods as well as from other hypnozoites within the same brood. We have previously estimated a lognormal distribution of relapse times for Papua New Guinea [4] using data from volunteers in US prisons [15, 16]. The volunteers were deliberately infected with the Chesson strain and the majority were given blood-stage treatment promptly [15]. The lognormal distribution was estimated to have a mean of 2.92 weeks and a standard deviation of 0.956 weeks, with a further 14 days from the primary infection until the beginning of the relapse time distribution. A very small proportion of relapses have very long times with this distribution: they are truncated at 550 days. Although additional estimates for the distribution of the time to the first relapse are available from data from malaria therapy patients and experimental studies [17-19], for the Chesson strain, the time to the first relapse is short and several distributions can fit well to this data thus providing very limited information on the distribution for the later relapses.

*(v) The cumulative number of primary infections since birth*

The mean number of cumulative primary infections since birth was estimated at the mid-point of each interval for each age-group, ITN use and village combination. We assumed that the seasonal pattern of primary infections repeats over time. We account for differential mosquito biting by age according to the median body surface area, as for (i). Due to seasonality, the number of cumulative primary infections can differ slightly across time intervals for the same age-group. We assume that ITN use has been the same since birth, as there have been no recent large-scale distribution campaigns in the area. The number of primary infections is used as a proxy for cumulative exposure, but this is indistinguishable from the number of broods in this analysis.

*(vi) Summary of inputs to the simulation model for the expected numbers of primary infections and relapses*

Table. Quantities in the simulation model for the expected numbers of primary infections and relapses

| Quantity | Description | value | Source |
| --- | --- | --- | --- |
|  |  |  |  |
| **Identifiers for covariate categories and time** | |  |  |
| $a$ | Category for combination of age-group, village, and ITN use^1^ | 28 categories (7 age-groups, 2 village, 2 ITN use) | - |
| $s$ | Five-day time-step of the simulation^2^ | Integer, $\geq0$ | - |
| $t$ | Two-month time interval of the cohort study^2^ | Integer, $\geq0$ | - |
|  |  |  |  |
| **Quantities from the previous analysis of the Ilaita cohort** | |  |  |
| $\lambda\left( a,s \right)$ | rate of primary infection for an individual in category $a$ at time-step $s$ | Numeric, $\geq0$ | Estimated in earlier work based on *P. falciparum* genotyping data for seasonality and *P. vivax* genotyping data for absolute incidence (i)[4]. Data on clinical illness are not used. |
| $m\left( a,s \right)$ | The number of primary infections for an individual in category $a$ introduced in time-step $s$ ^3^ | Integer, $\geq0$ | Follows Poisson distribution with mean $\lambda\left( a,s \right)$ |
|  |  |  |  |
| ***Incorporated in the previous analysis but from other data sources*** | | |  |
| $h_{i}$ | Number of relapses for brood $i$ | Sampled from distribution assuming a maximum of 15 | estimated in (iv) |
| $d$ | A constant used in the probability distribution for $h_{i}$ | 0.846^4^ or 0.926^5^ | estimated in (iv) |
| $\mu$  $\sigma$ | Mean of lognormal distribution for timing of relapse^4^  SD of lognormal distribution for timing of relapse | 2.92 weeks  0.956 weeks | Estimated in [4]  Estimated in [4] |
|  |  |  |  |
|  |  |  |  |
| **Additional inputs** | | |  |
| $v$ | Immune period after natural clearance (used for relapse classification B) | 20 days | Assumed |
| $q(a,s)$ | Incidence of treatment for an individual in category $a$ at time-step $t$ | Mean number of treatments | estimated from cohort data [4] |
| φ | Mean duration of blood-stage infection (Weibull distribution)  Scale parameter for Weibull distributions | 76 days^5^  27 days^6^  3.18 | M/therapy studies  Based on [5] |

^1^ Age-groups: 1-<1.5,1.5-<2,2-<2.5,2.5-<3,3-<3.5,3.5-<4,4 or more years. ITN use: use of ITN on <50% or $\geq$ 50% of nights

Village: Sunuhu, Ilaita

^2^ We convert between the five-day time-steps of the simulation and the two-month intervals of the cohort data

^3^Varies also by input parameter set

^4^ A further 14 days are added for the time from primary infection to the start of the relapse distribution

^5^ for input parameter value set 2 (longer mean blood-stage duration)

^6^ for input parameter value set 1 (shorter mean blood-stage infection)

It is possible to have more than one primary infection or relapse in the same five-day time-step. However, since the probabilities of illness are low in the age groups where this is most likely to occur, this has a negligible effect on the results.

**References**

1. Smith T, Ross A, Maire N, Chitnis N, Studer A, Hardy D, et al. Ensemble modeling of the likely public health impact of a pre-erythrocytic vaccine. PLoS Med. 2012;9:e1001157.

2. Tracton MS, Kalnay E. Operational ensemble prediction at the National Meteorological Center: practical aspects. Weather Forecast. 1993;8:378-98.

3. Penny MA, Verity R, Bever CA, Sauboin C, Galactionova K, Flasche S, et al. Public health impact and cost-effectiveness of the RTS,S/AS01 malaria vaccine: a systematic comparison of predictions from four mathematical models. Lancet. 2016;387(10016):367-75.

4. Ross A, Koepfli C, Schoepflin S, Timinao L, Siba P, Smith T, et al. The incidence and differential seasonal patterns of *Plasmodium vivax* primary infections and relapses in a cohort of children in Papua New Guinea. PLoS Negl Trop Dis. 2016;10(5):e0004582.

5. White M, Karl S, Koepfli C, Hofmann N, Felger I, Smith T, et al. *Plasmodium vivax* and *Plasmodium falciparum* infection dynamics: re-infections, recrudescences and relapses. Malar J. 2018;17:170.

6. Bretscher MT, Maire N, Felger I, Owusu-Agyei S, Smith T. Asymptomatic *Plasmodium falciparum* infections may not be shortened by acquired immunity. Malar J. 2015:294.

7. Felger I, Maire M, Bretscher M, Falk N, Tiaden A, Sama W, et al. The dynamics of natural *Plasmodium falciparum* infections. PloS one. 2012;7:e45542.

8. Snounou G, Pérignon JL. Malariotherapy - insanity at the service of malariology. Adv Parasitol. 2013;81:223-55.

9. Organization WH. WHO child growth standards. Available from: <http://www.who.int/childgrowth/standards/en/>.

10. Mosteller RD. Simplified calculation of body surface area. N Engl Med J. 1987;317:1098.

11. Karunajeewa HA, Mueller I, Senn M, Lin E, Law I, Servina Gomorrai P, et al. A trial of combination antimalarial therapies in children from Papua New Guinea. N Engl J Med. 2008;359(11):2545-57.

12. White NJ. Determinants of relapse peiodicity in *Plasmodium vivax* malaria. Malar J. 2011;10:297. doi: 10.1186/1475-2875-10-297.

13. Stadler E, Cromer D, Mehra S, Adekunle A, Flegg J, Anstey N, et al. Population heterogeneity in *Plasmodium viva*x relapse risk. PLOS Negl Trop Dis. 2022;16(12):e0010990.

14. White MT, Karl S, Battle KE, Hay SI, Mueller I, Ghani AC. Modelling the contribution of the hypnozoite reservoir to *Plasmodium vivax* transmission. eLife. 2014;10.7554:04692.

15. Coatney GR, Cooper WC, Young MD. Studies in human malaria. XXX. A summary of 204 sporozoite-induced infections with the Chesson strain of *Plasmodium vivax*. Journal National Malaria Society (US). 1950;9(4):381-96.

16. Coatney GR, Cooper WC. Studies in malaria VI. The organization of a program for testing potential antimalarial drugs in prisoner volunteers. Am J Hyg. 1948;47:113-9.

17. Lover AA, Coker RJ. Quantifying the effect of geographic location on epidemiology of *Plasmodium vivax* malaria. Emerg Inf Dis. 2013;19(7):1058-65.

18. Battle KE, Karhunen MS, Bhatt S, Gething PW, Howes RE, Golding N, et al. Geographical variation in *Plasmodium vivax* relapse. Malar J. 2014;13:144. doi: 10.1186/1475-2875-13-144

10.1186/1475-2875-13-144.</p>.

19. Lover AA, Zhao X, Gao Z, Coker RJ, Cook AR. The distribution of incubation and relapse times in experimental human infection with the malaria parasite *Plasmodium vivax*. BMC Infect Dis. 2014;14:539.
