# Supplementary material for: The probability of Plasmodium vivax acute illness following primary infection and relapse in Papua New Guinea
Source: PLoS Negl Trop Dis. 2025 Oct 3;19(10):e0013567. doi: 10.1371/journal.pntd.0013567 (PMC12510656; doi:10.1371/journal.pntd.0013567)
Supplement: S1 Text — (DOCX) [file pntd.0013567.s002.docx]

**S1 Text: The incidence of antimalarial treatment in the cohort**

The mean recorded number of anti-blood-stage malaria treatments dispensed per child per two-month interval did not vary substantially by age but did vary by season (Fig). The treatments may be given for *P. vivax*, for *P. falciparum,* which tends to occur in older children in this cohort, for other malaria species, mixed infections or given outside the study. Primaquine was not given.

| Fig. The mean number of anti-malarial treatments per child per interval by (a) age and (b) calendar time | |
| --- | --- |
| **** |  |

Open circles: mean number of treatments per two-month interval

Error bars: 95% confidence intervals
