# Supplementary material for: The probability of Plasmodium vivax acute illness following primary infection and relapse in Papua New Guinea
Source: PLoS Negl Trop Dis. 2025 Oct 3;19(10):e0013567. doi: 10.1371/journal.pntd.0013567 (PMC12510656; doi:10.1371/journal.pntd.0013567)
Supplement: S1 Fig — (DOCX) [file pntd.0013567.s003.docx]

|  |
| --- |

| **S1 Fig. The expected cumulative number of primary infections by age group and time interval** |
| --- |
| 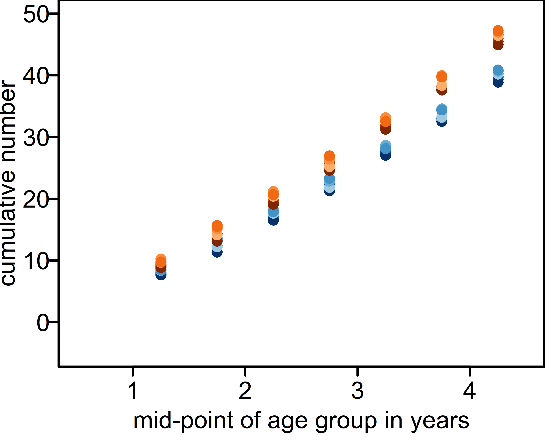 |
| Blues: Input parameter value set 2 with longer mean duration of blood-stage infection; Oranges: Input parameter value set 1 with shorter mean duration of blood-stage infection. The shade of colour represents the two-month interval by time from the onset of the transmission season from light to dark: the darkest shades represent children who reach the age-group at the end of the dry season. There is a very slight variation for the same age group at different time intervals due to the shift in the seasonal patterns from birth. The cumulative numbers shown are for ITN use less than 50% of nights and Ilaita village. |
