# Supplementary material for: The probability of Plasmodium vivax acute illness following primary infection and relapse in Papua New Guinea
Source: PLoS Negl Trop Dis. 2025 Oct 3;19(10):e0013567. doi: 10.1371/journal.pntd.0013567 (PMC12510656; doi:10.1371/journal.pntd.0013567)
Supplement: S1 Table — (DOCX) [file pntd.0013567.s004.docx]

|  |
| --- |

**S1 Table: Ability of the statistical model to recover known parameter values for the probabilities of illness following primary infection and relapse from simulated data**

S1 Table. Recovered point estimates for parameter^1^ values from 50 simulations (median, range)^2^

|  | $X^{*}$ | $p_{p0}$ | $p_{r_{1}0}$ | $p_{r_{2}0}$ |
| --- | --- | --- | --- | --- |
| **Values to recover 1** |  |  |  |  |
| True value | 0.05 | 0.8 | 0.5 | 0.2 |
| Model point estimates | 0.050 (0.045, 0.056) | 0.83 (0.63, 0.94) | 0.50 (0.32, 0.74) | 0.19 (0.10, 0.30) |
|  |  |  |  |  |
| **Values to recover 2** |  |  |  |  |
| True value | 0.05 | 0.3 | 0.1 | 0.05 |
| Model point estimates | 0.05 (0.04, 0.06) | 0.29 (0.17, 0.42) | 0.12 (0.06, 0.17) | 0.04 (0.02, 0.07) |
|  |  |  |  |  |
| **Values to recover 3** |  |  |  |  |
| True value | 0.2 | 0.8 | 0.5 | 0.2 |
| Model point estimates | 0.20 (0.17, 0.22) | 0.79 (0.45, 0.89) | 0.50 (0.33, 0.70) | 0.20 (0.08, 0.36) |
|  |  |  |  |  |
| **Values to recover 4** |  |  |  |  |
| True value | 0.2 | 0.3 | 0.1 | 0.05 |
| Model point estimates | 0.22 (0.19, 0.27) | 0.42 (0.23, 0.69) | 0.15 (0.05, 0.29) | 0.05 (0.01, 0.13) |

^1^The parameters are described fully in Table 2 and Methods of the main paper

^2^Median (range) of model point estimates from 50 simulated datasets using input parameter value set 1 and analyzed using model variant A2. The expected number of primary infections and relapses, and the treatment rates, were assumed to be those in the Ilaita cohort.
