# Supplementary material for: The probability of Plasmodium vivax acute illness following primary infection and relapse in Papua New Guinea
Source: PLoS Negl Trop Dis. 2025 Oct 3;19(10):e0013567. doi: 10.1371/journal.pntd.0013567 (PMC12510656; doi:10.1371/journal.pntd.0013567)
Supplement: Attachment — Submitted filename: response to reviewers.docx [file pntd.0013567.s006.docx]

The manuscript has not been sent for review: this page is included only to enable submission.
