# Supplementary material for: The probability of Plasmodium vivax acute illness following primary infection and relapse in Papua New Guinea
Source: PLoS Negl Trop Dis. 2025 Oct 3;19(10):e0013567. doi: 10.1371/journal.pntd.0013567 (PMC12510656; doi:10.1371/journal.pntd.0013567)
Supplement: Attachment — Submitted filename: response to reviewersR.docx [file pntd.0013567.s007.docx]

**Point by point responses to reviewers’ comments**

***Methods***

Reviewer #1: The author's methods are clear and can easily be repeated by anyone with knowledge in the field. The authors did a fantastic job accounting for the large variability associated with predictions of this nature by including several parameters and conditions for the simulations. The sample size is sufficient for a baseline prediction model and the statistics used are sound.

*Thank you for your comments.*

Reviewer #2: This paper tackles the important problem of assessing how relapses contribute to clinical episodes of P. vivax malaria. It has been hypothesized that relapses are less likely to cause clinical malaria than primary infections. Although this hypothesis is consistent with a number of experimental observations, and makes “biological sense”, it has not been definitively demonstrated. Furthermore, there are important implications for mathematical models of *P. vivax* transmission, as most published models to do not account for potential differences in the probability of illness between primary infections and relapses.

The underlying epidemiological data and genotyping data are solid. The modelling approach is appropriate, and the presented results are all credible.

*Thank you for your comments.*

Although I do not doubt any of the findings, my main concern relates to the presentation of the methods and results. Put simply, they’re very hard to follow.

For the benefit of the editorial team, I will lay out my understanding of the methodology (the authors should feel free to clarify any misunderstandings I may make). The starting data is from a cohort study in Ilaita in PNG. Blood samples from this study were genotyped using a single locus genotyping marker. These genotyping data were modelled to provide estimates of seasonally varying force of blood-stage infection over time, stratified by primary infection and relapse. This current paper builds on all of this past work and takes the previous model estimates of incidence as input. Furthermore, two different model estimates corresponding to different assumed duration of blood-stage infections are included.

Table 1 provides a description of 8 different models according to how relapses are defined, and assumptions regarding the probability that a relapse causes clinical illness.

The model also accounts for seasonality, age and covariates such as ITN use. A proxy of lifetime immunity is calculated by extrapolating backwards to estimate the total number of lifetime infections.

The OpenMalaria model is utilised to incorporate different model components and provide predictions of age-incidence curves. I really struggled to understand how the OpenMalaria model was used. When I follow the citation [39], it just says OpenMalaria and no other information is provided. OpenMalaria is a very impressive and well documented framework for mathematical modelling of malaria transmission, but to my knowledge there has been no published work on the use of P. vivax. Therefore, it’s not clear how it’s being used in this instance.

*We have rewritten the text to explain that the simple P. vivax model described in S1 appendix together with the estimates of clinical illness are embedded as a module within OpenMalaria (p8). This allows us to take advantage of OpenMalaria components for mosquito biting and an established machinery for generating predictions. The P. falciparum-specific modules are not used. The code and information on how to compile OpenMalaria with the vivax components is in the manuscript github repository (*[*https://github.com/rossaman4/vivax_clinical_illness*](https://github.com/rossaman4/vivax_clinical_illness)*).* *We recognize that this does not constitute a full vivax model since components are lacking, for example model components for patent and sub-patent infection and the decay of immunity. However these are not necessary for this particular paper.*

In summary, I have a very positive opinion of this important work, I am in agreement with the findings, but I have some substantial reservations about the presentation of the methods. A recommendation to the authors would be to simplify the presentation of the results by selecting one model variant and one set of model assumptions for the main manuscripts, and retain the others for the supplementary information.

*Thank you for the recommendation. We have tried to simplify the explanation of the different classifications of relapses (p5). We are hesitant to focus on only one model variant in the results section because it is not clear which to choose or how to give the other model variants equal billing. In the methods section, we describe the statistical model which estimates the probabilities of acute illness from the expected numbers of relapses and primary infections for one variant (A1) and then briefly describe how the model would vary for the other variants.*

The figures are also very hard to follow. They have multiple panels, different scales, no labels, and often no legends. The work as a whole would be easier to follow if the figures were simplified and made more consistent.

*We have improved the labelling of the figures (p19). We had inadvertently created some confusion because we included the figures in the text with legends and also as separate tiff files without legends. We have now resolved this.*

Reviewer #3: The methods of this study are sound and bolstered by a strong evidence base. The data from the cohort study and region is general is very rich.

*Thank you*

A minor question in SI section (iii) treatment - is the treatment time window sampled stochastically 2 weeks?

*The time of the treatment was sampled stochastically, but the prophylactic period was fixed at 14 days. We have edited the text to make this clearer.*

Main text classifying relapse - specify how early and late relapse are defined?

*We have clarified this (p5).*

Clarify the sentence that begins, "if there was selfing." Does the greatest genetic variation there refer to in the brood?

*We have clarified this sentence (p5). The primary infection is thought to contain greater genetic variation than each of the subsequent relapses of the same brood.*

***Results***

Does the analysis presented match the analysis plan?

-Are the results clearly and completely presented?

-Are the figures (Tables, Images) of sufficient quality for clarity?

Reviewer #1: All results are clearly labelled and illustrate the data in a logical way.

Reviewer #2: (No Response)

Reviewer #3: A minor suggestion to label plots (particularly Fig 1).

*We have now labelled all of the figures (p19).*

Fig 4 - in addition to this or a call out in the text about the proportion of symptomatic Pv illness due to relapse would also be useful for policy/advocacy purposes.

*We have mentioned the estimated overall contribution of relapses to clinical illness, and also the seasonal variation. Clearing (p11, p12, abstract)*

***Conclusions***

-Are the conclusions supported by the data presented?

-Are the limitations of analysis clearly described?

-Do the authors discuss how these data can be helpful to advance our understanding of the topic under study?

-Is public health relevance addressed?

Reviewer #1: The authors do a fantastic job at relating the findings to public health and are upfront with the limitations of their study. This is a great estimation in P. vivax relapse.

Reviewer #2: (No Response)

Reviewer #3: Yes- the conclusions are clear and it is very useful to have the findings of a modeling study anchored in the policy implications (ie, what groups to prioritize for treatment)

***Editorial and Data Presentation Modifications***

Reviewer #1: There are a few typos present in your manuscript, please double check the grammar prior to proof approval.

*We have checked the text for typos and grammar.*

Reviewer #2: (No Response)

Reviewer #3: I have provided minor modification suggestions above.

***Summary and General Comments***

Reviewer #1: (No Response)

Reviewer #2: (No Response)

Reviewer #3: The authors address an important topic using a complex but robust analytical framework and anchor it to the implications of policy.
