# Supplementary material for: The probability of Plasmodium vivax acute illness following primary infection and relapse in Papua New Guinea
Source: PLoS Negl Trop Dis. 2025 Oct 3;19(10):e0013567. doi: 10.1371/journal.pntd.0013567 (PMC12510656; doi:10.1371/journal.pntd.0013567)
Supplement: Attachment — Submitted filename: response to reviewers R3.docx [file pntd.0013567.s008.docx]

**Response to reviewers**

***Reviewer #2***: I will keep my comments here within the scope of my comments from my previous review. I previously wrote that I had a positive assessment of the importance of this work, and I maintain this opinion. My primary concern was that the models as presented in the manuscript were very difficult to understand, and I maintain this opinion.

In response to a recommendation that the authors clarify the description of the model methods, the authors have made some small edits on page 5.

*We thank Reviewer 2 for their comments. We would like to apologize and to say that there was no intention to treat their recommendations lightly. We know from our own experiences of reviewing that it is frustrating when authors do not address comments as fully as they might. In this case, we were unsure how to address the comments to clarify the text since we were uncertain which parts were unclear. We have now gone through the whole manuscript and Supporting Information again very carefully. We have checked the overall structure and then the text in each section. We checked each sentence for clarity and plain language. We removed any unnecessary words and read each section aloud to catch awkward phrases. Improvements have been made throughout and are documented in the tracked changes version. Although we hope now that each section is clear and the overall structure is coherent, it is true that this manuscript still requires more effort from the reader than would a simpler study requiring fewer components. However, we believe that it would not be possible provide estimates for primary infections and relapses separately with a simpler study.*

In response to the recommendation to “simplify the presentation of the results by selecting one model variant and one set of model assumptions for the main manuscripts, and retain the others for the supplementary information” the authors have chosen to retain all 8 model variants. This is their choice and I won’t argue further against it.

*We have edited the model variant section for clarity. We considered moving the alternative variants to supplementary information. However, this would either lead to figure 3 having variants that were not previously introduced, or it would place undue emphasis on one model variant. We would like to keep all the variants in this graph since these are the main results of this manuscript.*

In response to the comment about the figures being difficult to follow, the authors have made no change other than to copy and paste the existing figure legends to pages 19 and 20 of the revised manuscript. In the absence of any change, I maintain that the figures are still very difficult to understand.

*Our apologies - the original paper had missing figure legends for the figures at the end of the main manuscript, and we thought that this was what the reviewer was referring to.*

*There are five figures in the main manuscript, and three in the supplementary information. We have now carefully examined each one using the criteria: whether the figure was really needed, if the figure was mentioned in the most appropriate place in the text, if the title and footnotes were clear, that the axes were labelled properly and whether a simper figure could be devised.*

*We have made some changes:*

*Fig 1 (primary infection and relapse) – small change to title*

*Fig 2 (model fit) - change to text in the Results section where Fig 2 is mentioned; change to figure title and footnotes*

*Fig 3 (estimated probability of clinical illness following primary infection and relapse) – small changes to figure title and footnotes*

*Fig 4 (proportion of P. vivax illness due to primary infection) – re-designed for simplicity*

*Fig 5 (observed and predicted age-curves) – small change to main title and y-axis title*

*Supporting Information figures:*

*S1 (body surface area by age relative to a three-year-old child) - y-axis title shortened*

*S2 (reported number of anti-malarial treatments) – no change*

*S3 (cumulative number of primary infections) – y-axis title shortened*

In response to my comment on the difficulty of understanding the interaction with OpenMalaria, the authors have responded that more information is available at a github repository. However, reference 39 still just reads ‘OpenMalaria’, and the github repository is not cited anywhere in the manuscript – only in the response to reviewers. This is certainly not a helpful response.

*We agree that the github repository was not cited in the most helpful place – we had put it in the Data Availability section for the manuscript meta-data. We now cite the github repository under ‘Modelling Strategy’ in the Methods. The OpenMalaria reference now links to the OpenMalaria wiki which provides documentation.*

*The vivax components related to this manuscript have now been integrated as a module into the latest version of OpenMalaria (version 48). This avoids the need to swap in the new vivax module C++ files externally. We have re-run the results using this version and updated the text.*
